# A retrospective study to compare the clinical effects of individualized anatomic single- and double-bundle anterior cruciate ligament reconstruction surgery

**DOI:** 10.1038/s41598-020-71721-4

**Published:** 2020-09-07

**Authors:** Kang Chen, Weimin Zhu, Yizi Zheng, Fangjie Zhang, Kan Ouyang, Liangquan Peng, Haifeng Liu, Wenzhe Feng, Yong Huang, Greg Zhang, Zhenhan Deng, Wei Lu

**Affiliations:** 1 Department of Sports Medicine, Key Laboratory of Tissue Engineering of Shenzhen, Shenzhen Second People’s Hospital/The First Affiliated Hospital of Shenzhen University Health Science Center, Shenzhen, Guangdong China; 2 School of Medicine, Shenzhen University, Shenzhen, Guangdong China; 3 Guangzhou Medical University, Guangzhou, Guangdong China; 4 Clinical College of Anhui Medical University Affiliated Shenzhen Second People’s Hospital, Shenzhen, Guangdong China; 5 Department of Thyroid and Breast Surgery, Shenzhen Breast Tumor Research Center for Diagnosis and Treatment, National Standardization Center for Breast Cancer Diagnosis and Treatment, Shenzhen Second People’s Hospital/The First Affiliated Hospital of Shenzhen University Health Science Center, Shenzhen, Guangdong China; 6 Department of Emergency Medicine, Xiangya Hospital, Central South University, Changsha, Hunan China; 7 McGovern Medical School, University of Texas Health Science Center at Houston, Houston, TX USA

**Keywords:** Diseases, Medical research

## Abstract

To evaluate the clinical efficacy of single- and double- bundle individualized anatomic anterior cruciate ligament (ACL) reconstruction, we retrospectively analyzed the data and charts of 920 patients with ACL rupture who received individualized anatomic ACL reconstruction surgery at our center. All of the patients underwent arthroscopic ACL reconstruction with autologous hamstring tendons. The patients were divided into two groups: the single-bundle individualized anatomic reconstruction group (N = 539), and the double-bundle individualized anatomic reconstruction group (N = 381). The IKDC, Lysholm and Tegner scores were used to subjectively evaluate the function of the knee joint during the postoperative follow-up. The Lachman test, pivot shift test and KT-3000 were used to objectively evaluate the stability of the knee. All 920 patients participated in clinical follow-up (average duration: 27.91 ± 3.61 months) achieved satisfied outcomes with few complications. The postoperative IKDC, Lysholm and Tegner scores, and the objective evaluation of knee joint stability were significantly improved compared to the preoperative status in both groups (P < 0.05). No statistically significant difference was observed between the two groups at the final follow-up (P > 0.05). Therefore, no difference in terms of the IKDC, Lysholm and Tegner score, or KT-3000 was observed between the individualized anatomic single- and double-bundle ACL reconstruction techniques. Both techniques can be used to restore the stability and functionality of the knee joint with satisfactory short-term efficacy.

## Introduction

Anatomic single-bundle (SB) and double-bundle (DB) reconstruction are currently the most widely applied surgical treatments for anterior cruciate ligament (ACL) rupture^1^. Specifically, the ACL SB reconstruction procedure restores the anterior and posterior stability of the knee only by reconstructing the anteriormedial (AM) bundle; this traditional ACL reconstruction technique cannot restore knee kinematics and is associated with a reduced return to sport rate^2,3^. Many researchers believe that DB reconstruction, as the treatment closest to the original anatomical structure of the intact ACL, shows a biomechanical advantage^4,5^. This procedure has been reported to outperform SB reconstruction for better rotation stability of the knee joint and fewer graft failures^6^. However, several studies indicated that the number of bundles did not seem to influence clinical and subjective outcomes^7,8^. In addition, the drawbacks of DB reconstruction, such as extended operation time, high cost, increased risk of bone bridge fractures, and challenge in revision surgery, etc., also urge us to reconsider the necessity of this technique^9^.

In 2015, we proposed a concept of individualized DB ACL reconstruction^10^. With deepened understanding of the individual differences in ACL anatomic footprint, we further considered a number of indicators for the selection of appropriate surgical method^11^. We then retrospectively analyzed and compared the clinical outcomes between the arthroscopic SB and DB individualized anatomic ACL reconstruction with over two years of follow-up, in order to provide evidence for the DB individualized anatomic ACL reconstruction theory.

## Results

**Figure 1:**
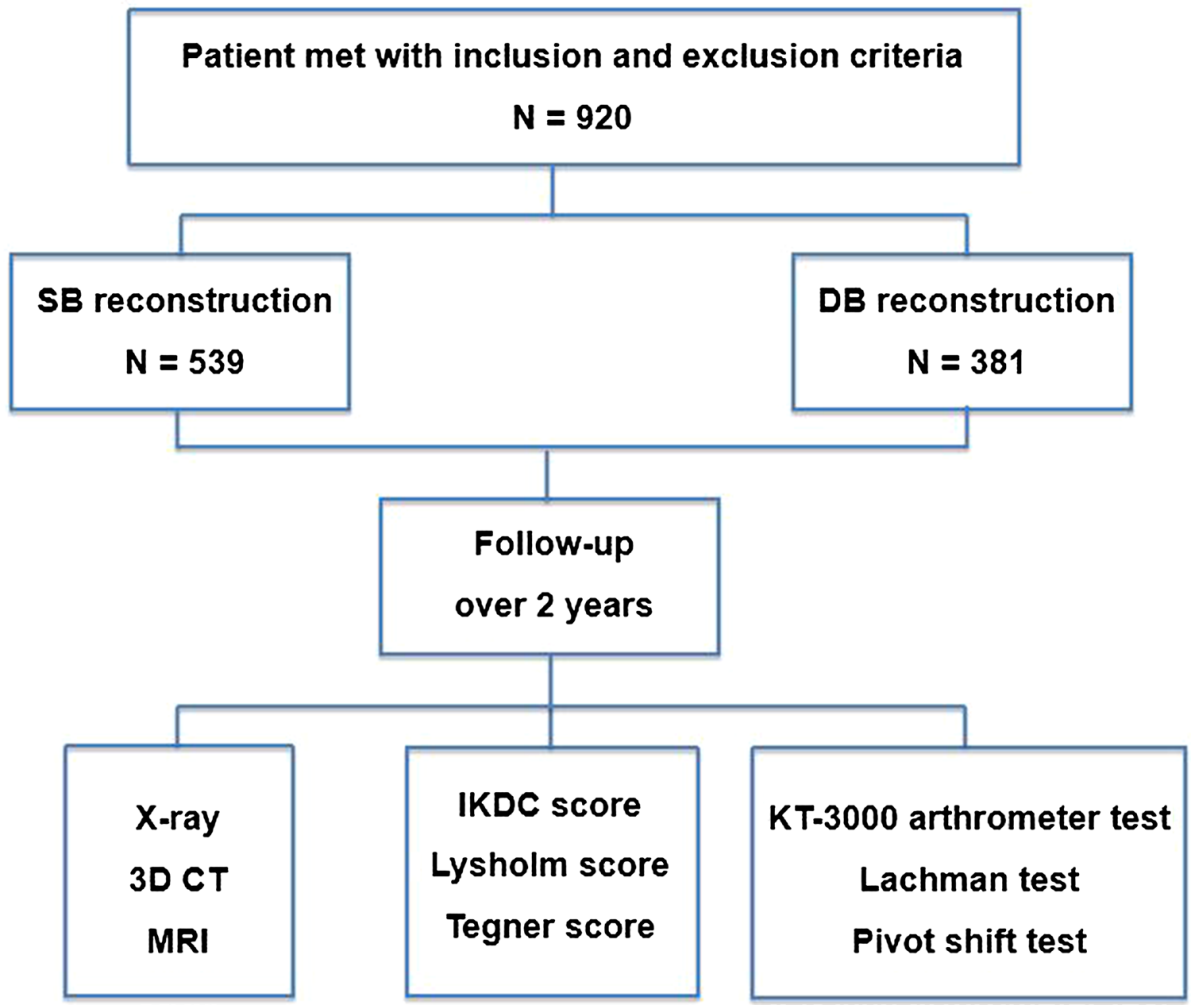
Flow diagram of the study design.

Flow diagram of the study design was presented in Fig. 1. The demographic characteristics of the patients are listed in Table 1. There was no statistical difference between the single-bundle reconstruction (SBR) group and double-bundle reconstruction (DBR) group in terms of gender, age, left and right knee, injury time interval and follow-up duration (P > 0.05).

**Table 1:** General information of patients.

Groups	Number	Gender		Age (years)	Side		Injury time (months)	Follow-up (months)
		Male	Female		Left	Right		
SBR	539	341	198	28.76 ± 7.25	209	330	17.65 ± 21.96	21.06 ± 1.59
DBR	381	221	160	27.76 ± 7.05	213	168	21.65 ± 28.71	24.28 ± 1.94
*t/χ2*		*χ*^*2*^ = 0.425		*t* = 0.647	*χ*^*2*^ = 0.751		*t* = 0.743	*t* = 0.735
*P*		0.503		0.455	0.367		0.452	0.712

*SBR* single-bundle reconstruction, *DBR* double-bundle reconstruction.

**Figure 2:**
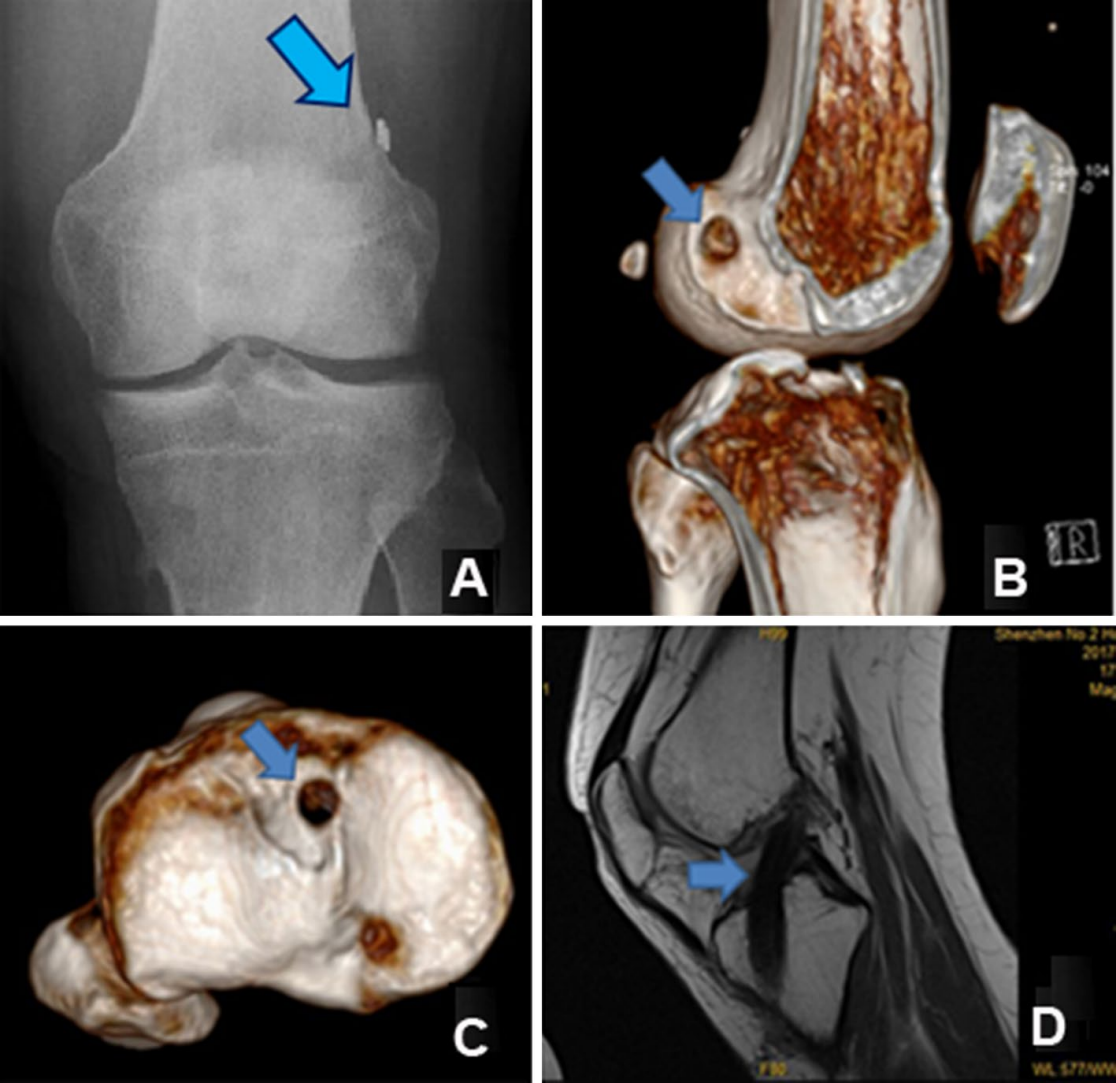
Postoperative radiological examination of single-bundle ACL individualized anatomical reconstruction after over 2 years. (**A**) X-ray: arrow shows the fixation of Endobutton. (**B**) 3D CT: arrow shows the femoral socket. (**C**) 3D CT: arrow shows the tibial socket. (**D**) MRI: arrow shows the graft.

**Figure 3:**
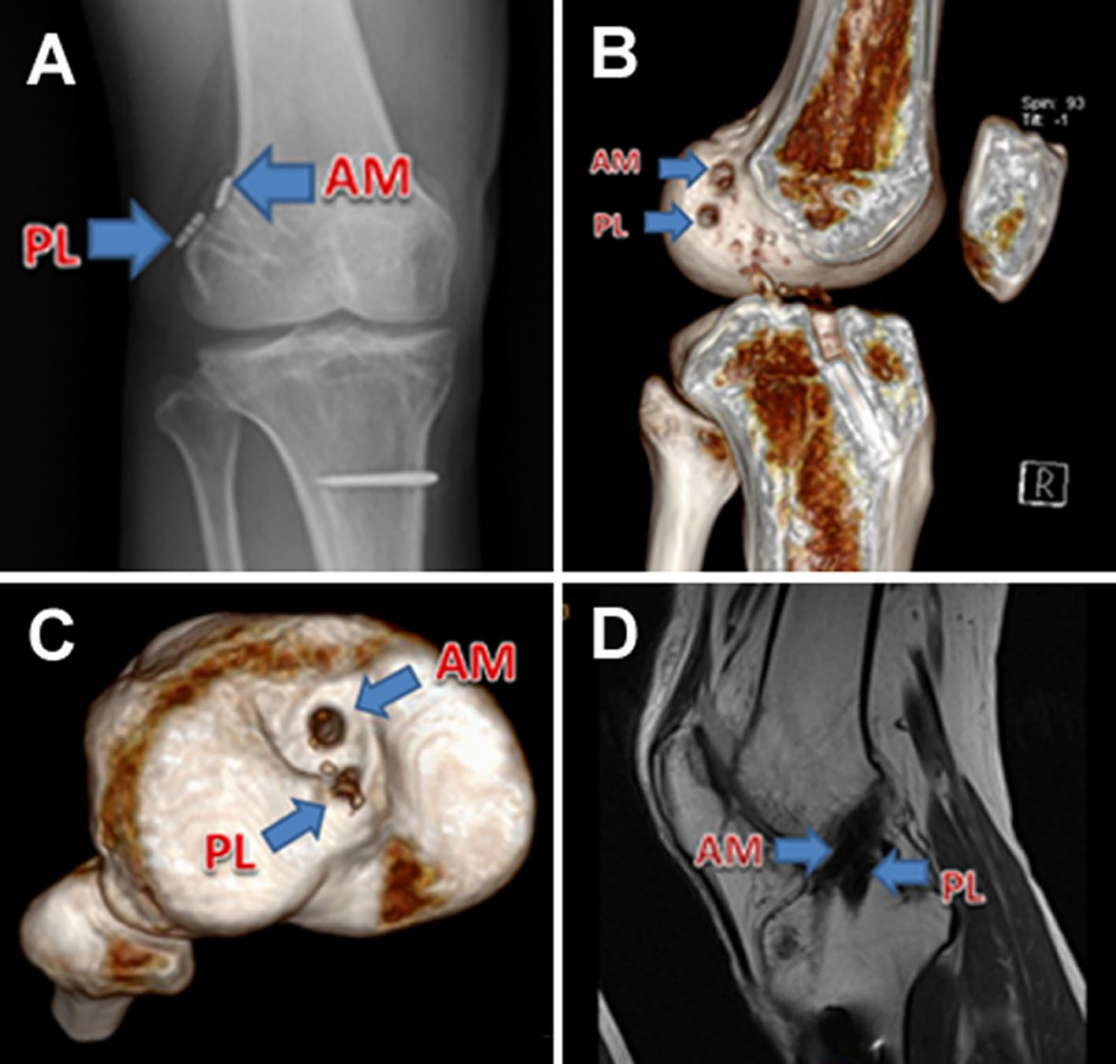
Postoperative radiological examination of double-bundle ACL individualized anatomical reconstruction after over 2 years. (**A**) X-ray: arrow shows the fixation of Endobuttons. (**B**) 3D CT: arrow shows the femoral socket. (**C**) 3D CT: arrow shows the tibial socket. (**D**) MRI: arrow shows the graft.

The follow-up duration ranged between 18 and 55 months with an average of 27.91 ± 3.61 months. All the patients recovered after surgical treatment without graft failure or deep venous thrombosis of the lower extremities. There was no statistically significant difference between the two groups in terms of complications (SBR group vs. DBR group, P > 0.05): knee joint infections (5 vs. 8), foreign-body reaction (4 vs. 3) and stiffness (4 vs. 3). The postoperative X-ray and three-dimensional computed tomography (3D CT) showed accurate bone tunnel and properly positioned screw, and magnetic resonance imaging (MRI) showed complete healing in both groups (Figs. 2, 3).

In the SBR group, 393 out of 539 patients managed to recover their pre-injury exercise level (excellent rate of 72.9%), and 119 patients were able to perform daily activities (e.g., walking, deep squatting, and walking up and down stairs) and mild physical activities. In the DBR group, 286 out of 381 patients recovered their pre-injury exercise level (excellent rate of 75%), and 76 patients were able to perform daily activities and mild physical activities. 27 patients from the SBR group and 19 patients from the DBR group reported occasional pain and residual subjective and objective joint instability.

The postoperative International Knee Documentation Committee (IKDC), Lysholm and Tegner scores were significantly improved in both groups compared to the pre-operative status (P < 0.01): IKDC scores increased from 59.03 ± 14.12 (40–77) to 89.40 ± 3.67 (83–95), Lysholm scores increased from 64.18 ± 19.11 (43–84) to 92.26 ± 5.12 (85–99), Tegner scores increased from 2.47 ± 0.82 (1–4) to 6.59 ± 0.51 (4–9) in the SBR group; IKDC scores increased from 60.15 ± 13.77 (42–76) to 90.03 ± 4.30 (83–96), Lysholm scores increased from 65.87 ± 18.94 (45–84) to 93.20 ± 4.83 (86–100), Tegner scores increased from 3.30 ± 1.27 (1–4) to 6.63 ± 0.79 (4–9) in the DBR group. But there was no significant difference between the two groups (P > 0.05, Table 2). In addition, no significant difference was found between the Lachman test and the pivot shift test according to the clinical examination at the final follow-up (P > 0.05, Table 3).

**Table 2:** Comparison of IKDC, Lysholm and Tegner scores before operation and at the last follow-up in both groups.

Groups	IKDC score		P value	Lysholm score		P value	Tegner score		P value
	Preoperative	Follow-up		Preoperative	Follow-up		Preoperative	Follow-up	
SBR	59.03 ± 14.12	89.40 ± 3.67	0.000	64.18 ± 19.11	92.26 ± 5.12	0.000	2.47 ± 0.82	6.59 ± 0.51	0.000
DBR	60.15 ± 13.77	90.03 ± 4.30	0.000	65.87 ± 18.94	93.20 ± 4.83	0.000	3.30 ± 1.27	6.63 ± 0.79	0.000
P value	0.672	0.630		0.593	0.602		0.572	0.697	

Postoperative follow-up compared with pre-treatment, P < 0.01, comparison between follow-up after treatment, P > 0.05.

*SBR* single-bundle reconstruction, *DBR* double-bundle reconstruction.

**Table 3:** Comparison of clinical findings between the two groups at the last follow-up.

Groups	Number	Lachman test		Positive rate	P value	Pivot shift test		Positive rate	P value
		(+)	(−)			(+)	(−)		
SBR	539	37	502	0.069	> 0.05	51	488	0.095	> 0.05
DBR	381	25	356	0.066		22	359	0.058	

The comparison between the follow-up groups after treatment, P > 0.05.

*SBR* single-bundle reconstruction, *DBR* double-bundle reconstruction.

The KT-3000 results of both groups showed that the difference between the affected and unaffected knee was significantly reduced after surgery (P < 0.01): in the SBR group decreased from 7.66 ± 6.32 (5.0–8.5) to 1.47 ± 1.15 (0.5–2.3); in the DBR group decreased from 6.82 ± 4.01 (5.0–8.2) to 1.40 ± 1.26 (0.5–2.1). No significant difference was found between the two groups (P > 0.05, Table 4).

**Table 4:** Comparison of KT-3000 results before operation and at the last follow-up in both groups.

Groups	30°of knee flexion		P value
	Preoperative	Follow-up	
SBR	7.66 ± 6.32	1.47 ± 1.15	0.000
DBR	6.82 ± 4.01	1.40 ± 1.26	0.000
P value	0.659	0.8002	

Postoperative follow-up compared with pre-treatment, P < 0.01, comparison between follow-up after treatment, P > 0.05.

*SBR* single-bundle reconstruction, *DBR* double-bundle reconstruction.

## Discussion

The results of our large-sample study with over 2 years of follow-up showed that the postoperative scores of IKDC, Lysholm and Tegner in both the SBR and DBR groups were significantly higher than the preoperative status, indicating that the knee joint function was recovered after SB or DB reconstruction. However, all the indicators showed no significant difference between the two groups at the final follow-up.

ACL cannot heal on its own after rupture. Reconstruction surgery under arthroscopy is the optimal method for treating ACL rupture at present^12^. The anatomic ACL reconstruction technique was proposed based on an extensive series of biomechanical, imaging, and clinical studies. This technique does not damage the inherent structure of the knee joint and is operated in accordance with the anatomical and biomechanical characteristics of the knee joint, which makes it more conducive to the recovery of the knee joint and the rotational stability than the traditional surgical method^13^.

Currently, there is a great deal of disagreement in the academic community regarding the choice of SB vs. DB reconstruction after ACL rupture. Meredick et al. reported that the SB reconstruction could restore most of the function (anterior–posterior stability) of the AM bundle, but not the posterolateral (PL) bundle. Meanwhile, it failed to restore the rotational stability as well, leading to relaxation in knee extension^14^. Freddie. Fu put forward the theory of maximizing the restoration of the ACL anatomical structure with DB reconstruction firstly in 2010^15^, the basic principle of which is to restore the size and shape of the patient’s ACL footprint to the best extent. DB reconstruction is to restore the AM and PL bundle simultaneously to ensure the alternating tension and relaxation of the two bundles during knee motion, which is close to the normal physiology^16^. The results of several biomechanical studies supported that the anatomic DB reconstruction could better restore the anterior–posterior and rotational stability of the knee, while SB reconstruction was not satisfactory in controlling the rotation and valgus torsion^17,18^.

However, other researchers found that under a simulated physiological load of quadriceps femoris, the DB reconstruction excessively limited the internal rotation of the tibia, which altered the normal trajectory of the patellofemoral joint, increased the contact pressure of the patellofemoral joint, and eventually led to cartilage damage^19,20^. In addition, many problems and difficulties may be encountered during the implementation of the DB reconstruction technique in clinical practice. Compared with SB reconstruction, the DB reconstruction technique is more time consuming and challenging for surgeons as it requires four bone tunnels and must be operated with a higher location accuracy. A larger amount of bone mass loss of the knee joint is not conducive to ACL reconstruction and revision, as the risk of lateral femoral condyle fracture and intraoperative bone bridge fracture between bone tunnels, as well as the probability of intercondylar fossa impaction will increase. An earlier study has also shown that it is difficult to establish a double bone tunnel for patients with smaller intercondylar fossa and shorter anteroposterior diameter of the tibia^21^. For these patients, SB reconstruction is a better choice due to the narrowness of the intercondylar fossa, the undersized area of the ligament stop point, and the finer diameter of the hamstring tendon. Since there is currently no objective and reliable method for detecting the stability of the knee joint rotation, the preferential results of DB reconstruction in restoring rotational stability remains to be further confirmed^21^. Our results were in line with previous studies. Kondo et al. reported that both SB and DB reconstructions could restore the knee joint anteroposterior and rotational stability with no significant differences in clinical outcomes^18^. Streich et al. found that both SB and DB achieved good clinical outcomes with no statistically significant differences in IKDC and Lysholm scores^22^.

The present study demonstrated satisfactory results in patients who received either SB or DB reconstruction based on a large sample with over 2 years of follow-up. From the perspective of anatomy and biomechanics, DB reconstruction is undoubtedly the best choice for completely restoring the normal function of the knee joint. Many indicators, including the size of the footprint, the shape of the femoral condyle as well as the AM and PL bundles, and the width of the intercondylar fossa, must be comprehensively considered. Individualized anatomic DB reconstruction can be chosen when the ACL footprints of the femur and tibia side are both greater than 14 mm and the width of the intercondylar fossa is greater than 12 mm. If such requirements are not fulfilled, individualized anatomic SB reconstruction is technologically friendlier, and can achieve equivalent clinical outcomes for most of the patients, especially those without special requirements on knee joint rotational stability. Our method of individualized anatomic DB reconstruction is proven to be able to achieve satisfactory clinical outcomes, but it is advisable to perform preoperative radiology evaluation and take accurate intraoperative measurements.

This study also presents with some limitations. Firstly, as a retrospective study, more prospective research including randomized controlled trials should be performed to provide stronger evidence. Secondly, the follow-up is relatively short, and long-term detection should be conducted in the future.

In conclusion, compared with SB individualized anatomic reconstruction, the DB individualized anatomic reconstruction does not exhibit any obvious advantage in terms of the postoperative IKDC score, Lysholm score, Tegner score and KT-3000 measurement of knee joint. The stability and functionality of knee joint can be well restored well in both groups with satisfactory short-term curative effect.

## Methods

### Patient recruitment

This retrospective study was approved by the ethics committee of Shenzhen Second People’s Hospital. All the participants had surrendered informed consent preoperatively. All methods were carried out in accordance with relevant guidelines and regulations. The patients who underwent primary ACL reconstruction at our department from October 2009 to May 2016 were reviewed. Patients would be included if they met the following criteria: (1) ≥ 18 years of age; (2) primary ACL surgery; (3) no concomitant ligament injury; (4) unilateral ACL injury; (5) no previous surgery on the affected knee; (6) no chondral lesion worse than Outerbridge grade 2; and (7) ACL rupture confirmed clinically and by MRI. The exclusion criteria included: (1) damage of multiple ligaments or injury of articular cartilage; (2) radiographic evidence of Kellgren-Lawrence grade 3 or 4 osteoarthritis and/or severe osteoporosis; (3) ACL injuries of both sides of the knees; (4) partial ACL rupture; (5) concomitant total or subtotal meniscectomy; or (6) young patients with unclosed growth plates. Overall, 920 patients were confirmed to meet all our inclusion criteria. The duration between injury and surgery ranged from 1 day to 8 years. All the included patients underwent both the initial surgery and individualized anatomic reconstruction. There were 667 patients with meniscus injury, for whom, the menisci were sutured, shaped, or resected according to the type of injury. All the surgeries were performed by the same senior surgeon. The patients were divided into two groups, the SBR group (N = 539) and the DBR group (N = 381).

### Surgical procedure

Every patient, regardless of group, was treated with combined spinal-epidural anesthesia. After placing the patient supine, the upper thigh was bound up with an inflatable tourniquet, and the surgical area routinely disinfected. Routine arthroscopy was performed in order to confirm the diagnosis of torn ACL.

The medial, lateral, and anterior internal approaches were adopted as described in previous literature^23^. Parameters including the length of ACL footprint, width of intercondylar nest, distance between bone tunnels, and distance between the bone and cartilage could be accurately measured and confirmed in all these approaches. A 3 cm longitudinal incision was created at the medial 1.5 cm of the tibial tubercle. Serving as grafts, the semitendinosus and gracilis tendons were exposed and harvested. For SB grafts, a double Krackow suture was performed with the EthiBond II non-absorbable line (Johnson & Johnson Medical N.V., Belgium) at both ends of the tendon. Then, the two sutured tendons were folded to create 4 strands. Finally, the diameter and length of the grafts were measured. The length was measured to be over 7 cm, and the diameter was 7–9 mm. For the DB grafts, a Double Krackow suture was performed with the EthiBond II non-absorbable line at both ends of the semitendinosus and gracilis tendons. Each sutured tendon was folded into 2 strands separately. The semitendinosus tendons were used as the AM bundles, and the gracilis tendons were used as the PL bundles. The diameter and length of the two grafts were measured respectively. In general, the AM bundle is over 6.5 cm in length and 6–7 mm in diameter, while the PL bundle is over 5.5 cm in length and 5–6 mm in diameter.

**Figure 4:**
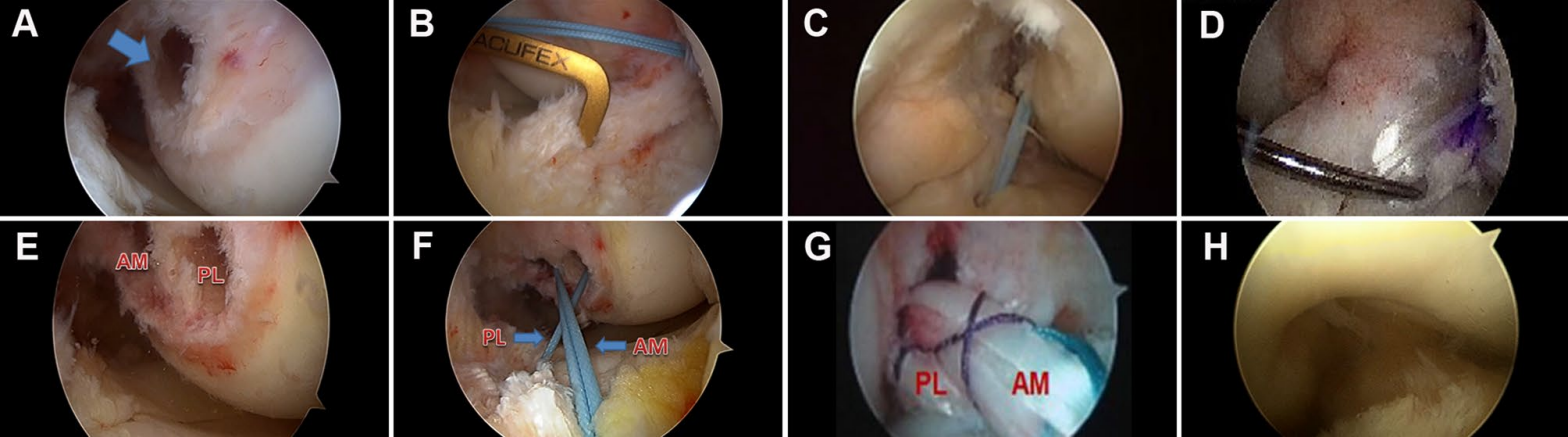
Surgical procedures of single- and double bundle ACL individualized anatomical reconstruction under arthroscopy. (**A**) Femoral insertion in SBR. (**B**) Tibial insertion in SBR. (**C**) Graft passed tibial tunnel in SBR. (**D**) Reconstructed ACL in SBR. (**E**) Femoral insertion in DBR. (**F**) Grafts passed tibial tunnel in DBR. (**G**) Reconstructed ACL in DBR. (**H**) Impingement test. *AM* anteromedial, *PL* posterolateral, *SBR* single-bundle reconstruction, *DBR* double-bundle reconstruction.

In accordance with the individualized anatomic reconstruction method proposed by Lu et al.^24^, the following principles are implemented: if there is a clear stump, the stump center is preferred as the center of the femur tunnel; if there is no stump or the stop point of the ligament is indistinguishable, the femur tunnel should be located according to the bony landmarks. The latter can be achieved by positioning the knee in 90° and observing from the anteromedial portal. The center of the bone canal was located at the lateral crest of the intercondylar fossa below the resident ridge. If the bony landmarks aren’t clear, it can be located between 30 and 35% below the lateral wall of the intercondylar fossa (Fig. 4A). However, the location of the tibial tunnel is usually determined by the center of ligament stump or the extension line of the lateral meniscus anterior angle (Fig. 4B). When a DB reconstruction is carried out, the length of the ACL footprint on the tibia side must be over 14 mm, and the width of the intercondylar fossa must be over 12 mm. If the ACL footprint completely disappears, both the anterior and posterior diameter of the resident ridge should be over 20 mm. If the measurement fails to meet any of the criteria above, DB reconstruction should not be performed. The detailed methods for creating femoral and tibial bone tunnels can be found in previous literature^24^.

The graft was first introduced into the tibial tunnel with a guide wire. In SB reconstruction, the graft was pulled directly into the femoral tunnel and fixed on the femoral side by flipping over the Endobutton (Smith & Nephew, MA). Then on the tibial side, the graft was manually tightened and fix at 30° knee flexion by a door-shaped nail (2.3 mm Kirschner’s needle). Subsequently, the fixation needed to be further strengthened using a hydroxyapatite interference screw with a diameter of 1 mm larger than the graft (Fig. 4C,D).

In the DB reconstruction, after creating the femoral tunnels (Fig. 4E), the AM and PL grafts were firstly passed through the respective tibial tunnels (Fig. 4F) and then pulled out of the femoral tunnels. The femoral side was fixed by Endobutton suspension. On the tibial side, the graft sutures were tightened and knotted separately. A 2.3 mm Kirschner needle was used to fix the grafts (AM fixed at 30° flexion and PL at the full extension position), which were then reinforced using an interference screw with a diameter of 1 mm larger than the grafts (Fig. 4G).

Following the ACL reconstruction, flexion of the knee joint from 90° to full extension was performed in order to observe whether the femoral condyle and posterior cruciate ligament demonstrated any impingement (Fig. 4H).

### Postoperative treatment and rehabilitation

All patients were treated with 1 g cefoxitin BID for 48 h to prevent infection, and the affected limb was wrapped with cotton pad for 3 days. X-ray and 3D CT were performed immediately after surgery to evaluate the bone tunnel and fixation, and MRI was performed to check the ligament healing at 3, 6, 12 and 24 months after surgery respectively.

The same postoperative rehabilitation plan was implemented for the patients in both groups. Specifically, the affected limb was immobilized with adjustable support. On the second day postoperatively, the patients were requested walk with crutches under protection of knee braces (the specific walking time was determined based on whether the meniscus was sutured). The patients were encouraged to flex their knees from 0 to 90° within the second to the fourth week, and up to 120° within the sixth to the eighth week. However, they were instructed not to flex the affected knee over 120° in the first 3 months postoperatively. The braces were used for at least 2 months. Six patients were allowed to swim and cycle 6 months after the operation, to begin jogging 10 months after the operation, and to participate in strenuous exercises 18 months after the operation^25^.

### Observation indicators

Knee function was assessed by IKDC, Lysholm and Tegner scores before operation and during the follow-up. The preoperative and follow-up KT-3000 arthrometer (MEDmetric, San Diego, CA, USA) measurements of the tibial anterior displacement were taken by applying a tension of 134 N with knee joint flexion at 30°. The Lachman test and pivot shift test were performed to evaluate the knee joint stability. A positive Lachman test is defined as: with the knee flexed 20°-30°, the tibia is displaced anteriorly relative to the femur with either a soft endpoint or a displacement greater than 4 mm. The pivot shift test is considered positive if the proximal tibia subluxes anteriorly on the distal femur at about 30° of flexion.

### Statistical analysis

The study data was expressed as mean ± standard deviation (SD) and analyzed by SPSS 18.0 software (SPSS Inc., Chicago, IL, USA). Independent samples t-test and χ^2^ test were done on the general data of the patients. The preoperative and postoperative IKDC, Lysholm, Tegner scores, and KT-3000 measurements were tested for paired t-test. Chi-squared test was used for the analysis of Lachman and pivot shift tests. The threshold for statistically significant differences is defined as P < 0.05.

## References

[1] Sundemo D (2018). Increased postoperative manual knee laxity at 2 years results in inferior long-term subjective outcome after anterior cruciate ligament reconstruction. Am. J. Sports Med..

[2] Ardern CL, Taylor NF, Feller JA (2014). Fifty-five per cent return to competitive sport following anterior cruciate ligament reconstruction surgery: an updated systematic review and meta-analysis including aspects of physical functioning and contextual factors. Br. J. Sports Med..

[3] Legnani C (2019). Return to sports and re-rupture rate following anterior cruciate ligament reconstruction in amateur sportsman: long-term outcomes. J. Sports Med. Phys. Fitness..

[4] Svantesson E (2017). Double-bundle anterior cruciate ligament reconstruction is superior to single-bundle reconstruction in terms of revision frequency: a study of 22,460 patients from the Swedish National Knee Ligament Register. Knee Surg. Sports Traumatol. Arthrosc..

[5] El-Sherief F (2018). Double-bundle anterior cruciate ligament reconstruction is better than single-bundle reconstruction in terms of objective assessment but not in terms of subjective score. Knee Surg. Sports Traumatol. Arthrosc..

[6] Järvelä S (2017). Double-bundle versus single-bundle anterior cruciate ligament reconstruction: a prospective randomized study with 10-year results. Am. J. Sports Med..

[7] Aga C (2018). No difference in the KOOS quality of life subscore between anatomic double-bundle and anatomic single-bundle anterior cruciate ligament reconstruction of the knee: a prospective randomized controlled trial with 2 years' follow-up. Am. J. Sports Med..

[8] Adravanti P (2017). Single-bundle versus double-bundle anterior cruciate ligament reconstruction: a prospective randomized controlled trial with 6-year follow-up. J. Knee Surg..

[9] Tajima T (2019). Early weight-bearing after anterior cruciate ligament reconstruction with hamstring grafts induce femoral bone tunnel enlargement: a prospective clinical and radiographic study. BMC Musculoskelet. Disord..

[10] Lu W (2015). Femoral footprint variation of the posterolateral bundle of the anterior cruciate ligament and double-bundle reconstruction. Knee..

[11] Shen W, Fu FH (2008). Anterior cruciate ligament insertion site anatomy. Arthroscopy.

[12] Chhabra A (2006). Anatomic, radiographic, biomechanical, and kinematic evaluation of the anterior cruciate ligament and its two functional bundles. J. Bone Joint Surg. Am..

[13] Fukuda TY (2013). Open kinetic chain exercises in a restricted range of motion after anterior cruciate ligament reconstruction: a randomized controlled clinical trial. Am. J. Sports Med..

[14] Meredick RB, Vance KJ, Appleby D, Lubowitz JH (2008). Outcome of single-bundle versus double-bundle reconstruction of the anterior cruciate ligament: a meta-analysis. Am. J. Sports Med..

[15] Schreiber VM, van Eck CF, Fu FH (2010). Anatomic double-bundle ACL reconstruction. Sports Med. Arthrosc. Rev..

[16] Tsai AG, Wijdicks CA, Walsh MP, Laprade RF (2010). Comparative kinematic evaluation of all-inside single-bundle and double-bundle anterior cruciate ligament reconstruction: a biomechanical study. Am. J. Sports Med..

[17] Petersen W (2007). Biomechanical evaluation of two techniques for double-bundle anterior cruciate ligament reconstruction: one tibial tunnel versus two tibial tunnels. Am. J. Sports Med..

[18] Kondo E, Yasuda K, Azuma H, Tanabe Y, Yagi T (2008). Prospective clinical comparisons of anatomic double-bundle versus single-bundle anterior cruciate ligament reconstruction procedures in 328 consecutive patients. Am. J. Sports Med..

[19] Gadikota HR (2009). Biomechanical comparison of single-tunnel-double-bundle and single-bundle anterior cruciate ligament reconstructions. Am. J. Sports Med..

[20] Yoo JD (2005). The effect of anterior cruciate ligament reconstruction on knee joint kinematics under simulated muscle loads. Am. J. Sports Med..

[21] Zhou JB (2011). Anatomic anterior cruciate ligament reconstruction: a surgical technique more original than traditional. Chin. J. Sports Med..

[22] Streich NA, Friedrich K, Gotterbarm T, Schmitt H (2008). Reconstruction of the ACL with a semitendinosus tendon graft: a prospective randomized single blinded comparison of double-bundle versus single-bundle technique in male athletes. Knee Surg. Sports Traumatol. Arthrosc..

[23] Cohen SB, Fu FH (2007). Three-portal technique for anterior cruciate ligament reconstruction: use of a central medial portal. Arthroscopy.

[24] Lu W, Wang D, Xiao D (2013). Clinical study of anterior cruciate ligament in situ double-bundle reconstruction. Chin. J. Joint. Surg..

[25] Zhu W, Wang D, Han Y, Zhang N, Zeng Y (2013). Anterior cruciate ligament (ACL) autograft reconstruction with hamstring tendons: clinical research among three rehabilitation procedures. Eur. J. Orthop. Surg. Traumatol..

